# Evaluation of Superior Semicircular Canal Morphology and Its Relationship with Glenoid Fossa Roof Thickness Using Cone Beam Computed Tomography

**DOI:** 10.1155/2022/1565038

**Published:** 2022-12-17

**Authors:** Bahare Davvaz, Mahvash Hasani, Abdolaziz Haghnegahdar

**Affiliations:** ^1^Student Research Committee, Dental School, Shiraz University of Medical Sciences, Shiraz, Iran; ^2^Department of Oral and Maxillofacial Radiology, Dental School, Shiraz University of Medical Sciences, Shiraz, Iran

## Abstract

**Purpose:**

This study aimed to evaluate the bone thickness of the superior semicircular canal (SSC) roof and its relationship with the roof thickness of the glenoid fossa (RGF).

**Methods:**

The cone beam computed tomography (CBCT) images of 280 patients (560 temporal regions) were surveyed. The lowest thickness of the SSC roof was measured and categorized based on the radiological patterns of the Cisneros et al. classification. The thickness of GF and the presence of dehiscence in this part were determined, as well. The relationship between the thickness of the GF roof and the bone thickness covering the SSC was also assessed.

**Results:**

The mean thickness of the SSC roof was 0.93 ± 0.48 mm, with no significant difference among different age groups and genders (*p* > 0.05). However, superior semicircular canal dehiscence (SSCD) was more prevalent among females over 45 years old. Similarly, the individuals with the dehiscence of the GF roof had a 12.93-fold higher chance of SSCD development.

**Conclusions:**

The results indicated that the thickness of the bone overlying the SSC was significantly related to the roof thickness of the GF. However, an increase in age resulted in no significant change in the bone thickness of the SSC roof. Gender also had no role in changing the thickness of the bone overlying the SSC. Considering the decrease in the thickness of the SSC roof among females over 45 years of age, menopause may be responsible for this occurrence as well as for the increase in the prevalence of SSCD.

## 1. Introduction

The semicircular canals are a part of the vestibular system including superior, posterior, and horizontal ducts. The superior semicircular canal (SSC) is located perpendicular to the axis of the petrous bone and a segment of SSC is closely related to arcuate eminence [1]. Tinny openings in the bone covering the SSC may lead to a superior semicircular canal dehiscence (SSCD) syndrome. Minor et al. first explained this syndrome in 1998 [2]. There are some theories explaining the etiology of SSCD, but the exact reason has yet to remain undetermined. Because of inherited factors in some patients, the bone in that area is not thick enough in 1-2% of the world's population. As a result, head injury, cerebrospinal fluid (CSF) flow, and changes in intracranial pressure can disrupt this thin layer. Therefore, a congenital thin bony layer can trigger the onset of symptoms in adults [2–4]. Lack of bone reduces the stimulus threshold and causes the system to be sensitive to sound and alteration in the middle ear pressure, which can induce vertigo, eye movements, hearing loss, autophony, and a feeling of aural fullness [2].

In the past, conventional Computed Tomography (CT) images were used to evaluate the SSCD [3, 5–12]. In 2011, Sequeira et al. reported that CT scans could not be used as an exclusive gold standard for SSCD [11]. Hence, Cone Beam CT (CBCT) is currently recommended due to such advantages as a lower radiation dose and a thinner slice. Reconstruction of high-resolution three-dimensional images is also possible with this new imaging technology [13, 14]. The diagnosis of an SSCD syndrome must be made based on both radiological findings and clinical examination, because some patients have vestibular or auditory symptoms suggestive of SSCD and yet don't present radiological dehiscence, which is essential to make the diagnosis or, conversely, are free of any symptoms while showing SSCD on CBCT imaging [10, 15–17].

Some studies conducted in the West confirmed that the thickness of the bone covering the SSC decreased with increasing age [18, 19]. However, in a study performed on Chinese descent, no significant age-related change was observed in bone thickness [20]. Additionally, SSCD was more prevalent amongst Asian females who had osteoporosis [21]. Crovetto et al. also stated that SSCD was more common in menopausal women [18]. Moreover, just two studies found a correlation between the roof thickness of the glenoid fossa and the thickness of the bone masking the SSC [22, 23]. Glenoid fossa (GF) refers to a depression in the temporal bone that articulates with the mandibular condyle. The roof of GF is a thin plate of bone placed between the temporomandibular joint (TMJ) complex and the middle cranial fossa [22]. Kurt et al. indicated that patients with TMJ symptoms were at a higher risk of SSCD [14].

The current study aims to evaluate the relationship between the thickness of the bony overlying of the SSC and the RGF using CBCT with a larger sample size than the previous study. Besides, it determines the association between the thickness of the SSC bone and gender as well as age. It is noticeable that only a few studies have described an association between SSCD and the roof thickness of the GF and no numerical relationship has been reported in them.

## 2. Materials and Methods

The study was accepted by the Human Ethics Review Committee of Shiraz Dental School (# IR Sums.Dental.REC.1400.034). The study population included all CBCT images available in the archives of the radiology department of Shiraz Dental School. All CBCT images showing the target area completely and readably could be included in the study. However, the images that were outside the target area or had low quality, bone diseases, and previous surgical procedures were excluded. Written informed consent forms for the usage of the images were obtained from the patients. The sample size was calculated as 560 temporal regions (280 patients) based on the expected prevalence of SSCD reported in the literature (approximately 10%), *d* = 2.5%, and a confidence level of 95%.

**Figure 1:**
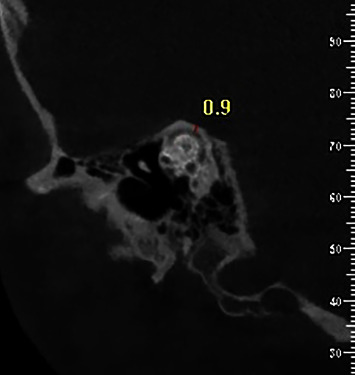
The thinnest part of the superior semicircular canal (SSC) coverage was measured as the SSC thickness.

**Figure 2:**
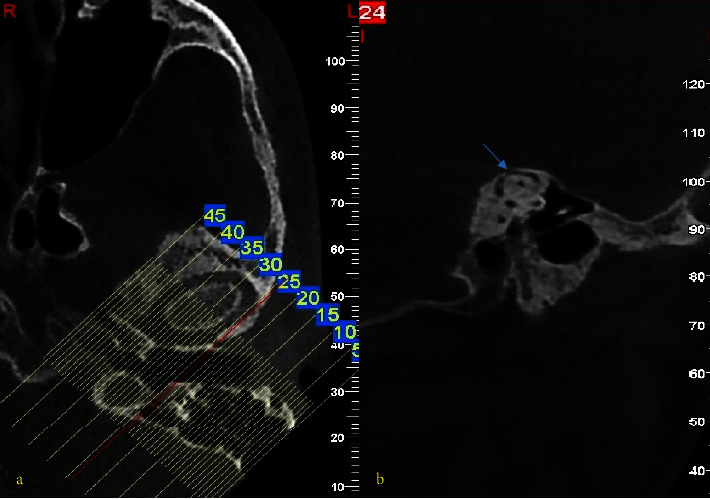
Pöschl€ transverse sectioning to depict superior semicircular canal (SSC) (a) and visualize SSC with a dehiscent roof (blue arrow) (b).

The CBCT images were prepared using NewTom VGi (QR-SRL, Venora, Italy) with a voxel resolution of 0.3 mm (8 mA, 110 kVp), a 3.6-second scan, and a field of view of 15 × 15 and were transferred to the NNTviewer software (NewTom, Venora, Italy). All reconstructions were prepared with a thickness of 1 mm and at a 1-mm distance. Under standard viewing conditions, all images were reviewed by authors on the presence or absence of SSCD as well as the thickness of the bone covering the semicircular canal. The images were reformatted into the Pöschl transverse pyramidal plane for an easier and more reliable assessment of SSCD [14, 22]. In these planes, SSC seemed like a ring. The thinnest part of the bone masking the SSC was measured using a digital ruler (Figures 1 and 2).

**Figure 3:**
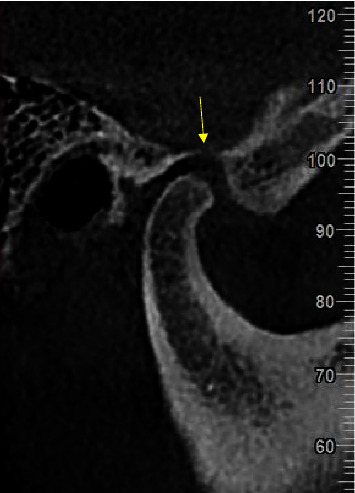
Dehiscence of the roof glenoid fossa (RGF) on the sagittal section (yellow arrow).

The obtained measurements along with the age and sex of the patients were recorded anonymously in the relevant tables. The thinnest part of the GF roof in sagittal sections was considered the RGF. The presence of any dehiscence in the GF roof was also recorded (Figure 3).

The patients were divided into six groups based on age: <20 years, 20–29 years, 30–39 years, 40–49 years, 50–59 years, and ≥60 years. These age groups were compared with respect to morphology and roof thickness. To assess the impact of menopause on the bone thickness of SSC, the male and female participants were divided into two age groups; i.e., under 45 and over 45 years, according to the research by Crovetto et al. [18]. The male and female participants in these two age groups were compared regarding the mean thickness of the SSC bone. According to Cisneros et al., the SSC bone thickness was categorized into five patterns including normal pattern (0.6–1.7 mm), thick pattern (≥1.8 mm), papyraceous pattern (≤0.5 mm), pneumatized pattern (multiple supra labyrinthine cells like a woven structure), and dehiscence pattern, in which the continuity of the roof cortices was lost [24]. Akay et al. also classified RGF into three groups, namely less than 1 mm, between 1 and 2 mm, and more than 2 mm. Logically, the absence of the GF roof bone was considered GF dehiscence [23].

### 2.1. Statistical Analysis

The Chi-square test, odds ratio (OR), and the corresponding 95% confidence interval (CI) were used to assess the relationship between SSCD and other variables. Additionally, one-way ANOVA and Pearson's correlation coefficient were employed to assess the relationship between age and the thickness of the bone overlying the SSC. Besides, mean, standard deviation (SD), frequency, and percentage were used to describe the data. All statistical analyses were performed using the IBM SPSS version 22.0 for Windows (SPSS Corp, Armonk, NY, USA), and *p* < 0.05 was considered statistically significant.

## 3. Results

In this retrospective study, 280 CBCT scans (560 temporal bones) including 122 males (43.6%) and 158 females (56.4%) were assessed. The mean age of the participants was 31.40 ± 12.80 years, ranging from 7 to 74 years. Among females, 137 patients (86.7%) were 45 years old and below, while 21 patients(13.3%) aged above 45 years. Among males, on the other hand, 105 patients (86.1%) were 45 years old and below, while 17 patients (13.9%) aged above 45 years.

The mean thickness of the SSC bone was 0.93 ± 0.48 mm in the study sample. The results showed no significant difference among the age groups concerning the mean thickness of the SSC roof (*p*=0.660) (Table 1).

Moreover, there was no significant correlation between age and the thickness of the SSC roof (*r* = −0.04, *p*=0.414). The results also showed no significant difference between the two genders in terms of the thickness of the SSC roof (*p*=0.419).

Based on Cisneros et al.' [24] classification, 408 cases (72.9%) had the “normal” pattern, 34 cases (6.1%) had the “papyraceous” pattern, 37 cases (6.6%) had the “thick” pattern, and 38 cases (6.8%) had the “pneumatized” pattern. In addition, the dehiscent pattern was detected in 43 cases (7.7%) (Table 2).

The results indicated no significant relationship between gender and the existence of SSCD (*p*=0.933). In other words, males and females had the same chance of developing SSCD (OR = 1.02, 95% CI: 0.54–1.92). Nevertheless, the females aged over 45 years were 3.79 times more likely to have SSCD compared to younger females (OR = 3.79, 95% CI: 1.51–9.53) (Table 3).

The mean RGF was 0.75 ± 0.51 mm. Based on the results of the chi-square test, OR, and the corresponding 95% CI, the relationship between the SSCD and the GF roof dehiscence was statistically significant (*p* ≤ 0.001). Accordingly, the cases with GF roof dehiscence were 12.93 times more likely to have SSCD compared to those without GF roof dehiscence (OR = 12.93, 95% CI: 6.59–25.37). Furthermore, a relationship was detected between SSCD and RGF thickness. Accordingly, the thicker the GF roof, the lower the chance of SSCD would be (*p*=0.008) (Table 4).

## 4. Discussion

SSCD has been defined as the absence of the bone covering the SSC. Timely diagnosis of SSCD is essential due to a variety of inner ear complications such as pressure-induced vertigo, aural fullness, autophony, and hearing loss [2]. Three-dimensional radiological findings can play a key role in SSCD diagnosis. Some experts have confirmed the superiority of CBCT over CT scans for temporal imaging. There are many studies in the literature concerning the detection of SSCD using CT scans [3, 5–12]. However, just a few studies have been conducted on SSCD diagnosis by CBCT imaging [14, 20, 23, 25, 26]. Dalchow et al. concluded that CBCT had the potential to be considered a complete imaging technique for assessing SSC in patients with otologic problems [25]. Moreover, Mahulu et al. realized that CBCT could obviously show the internal structure of the ear and help verify SSC [20]. Overall, CBCT has many advantages over CT scans including a lower effective radiation dose and easier image acquisition. In addition, multiplanar reconstruction of high-resolution three-dimensional images is possible in CBCT [11, 14]. Hence, maxillofacial radiologists can assist otolaryngology specialists in examining the temporal bone using CBCT images.

Generally, congenital and acquired factors may thin or disrupt the bone covering the SSC [27]. However, controversial information is available regarding the main cause of SSCD. Some researchers believe that congenital causes are more important compared to acquired ones [3, 28–30]. For instance, Carey et al. concluded that owing to congenital factors, the bone masking the SSC is thin at birth and begins to thick after the age of three years [3]. In addition, Takahash et al. stated that a possible cause of SSCD might be the anatomical feature of the middle cranial fossa in the fetus [29]. On the other hand, some researchers have disclosed that SSCD results more frequently from acquired conditions. In this context, aging, menopause, trauma, and osteoporosis have been mentioned as the acquired factors contributing to the thinning of the bone overlying the SSC [18–20, 23, 31–33]. Western studies have revealed a significant difference between old and young age groups regarding bone thickness [18, 19, 31, 32]. Nadgir et al. found an increased prevalence of radiographic SSCD with an increase in age [31]. Davey et al. also emphasized that the thickness of the SSC roof decreased in elderly individuals, and confirmed that each individual would lose about 0.005 mm of the bony layer of the SSC each year [19]. In contrast, the studies carried out in Asia did not approve of the relationship between SSC bone thickness and advancing age [20, 23, 33]. For instance, Akay et al. found no significant changes in bone thickness with an increase in age [23]. The current study findings also demonstrated no significant difference between young and old patients with regard to the thickness of the bone masking the SSC. The difference between European and American societies and Asian ones may be attributed to ethnic factors, different types of diet, and geographical location.

The present study results revealed no statistically significant difference between the two genders in terms of the thickness of the SSC roof, which was in agreement with the results of the studies by Akay et al. [23] and Evlice et al. [33]. Nonetheless, Karimnejad et al. reported that SSCD was 1.2 times more prevalent amongst females based on a review of publications between 2000 and 2015 [34]. This discrepancy might be associated with different races under investigation.

Osteoporosis and menopause can be considered as other factors involved in the development of SSCD. Yu et al. indicated that SSCD was more prevalent among Asian women who had osteoporosis [21]. Moreover, Crovetto et al. showed that women aged over 40 years lost about 0.10 mm of the bone covering the SSC, that implied menopause could cause SSCD [18]. However, Mahulu et al. reported an insignificant difference between males and females over 45 years old and those below 45 years of age regarding the thickness of the SSC bone [20]. In the present research, a significant change was observed in the SSC thickness among the females aged 45 years or below compared to those over 45 years old, but no significant change was detected among the males aged 45 years or below in comparison to those over 45 years old. Additionally, the females aged over 45 years were 3.79 times more likely to have SSCD compared to the males aged over 45 years. These results suggest the consideration of the impact of menopause and osteoporosis while assessment of SSCD in further studies.

In the study executed by Cisneros et al., the papyraceous pattern was the most prevalent pattern after the normal thickness of the SSC roof [24]. In the current investigation, however, the incidences of different patterns were almost the same, except for the normal pattern. The prevalence of SSCD has been reported in various articles mostly using CT scans [10, 21, 31]. For example, Nadgir et al. investigated 306 temporal regions and reported a prevalence of 7% for SSCD [31]. Yu et al. also evaluated 496 patients in the neurology clinic in a community health center and stated that the prevalence of SSCD was 6.6% [21]. Moreover, two large series found that the incidence of SSCD ranged from 4% to 8% [10]. Noticeably, a few studies have measured the prevalence of SSCD using CBCT [14, 23]. In this study using CBCT images, the prevalence of SSCD was 7.7%. This result was similar to the findings of the studies benefitting from CBCT images such as the one conducted by Kurt et al. (6.28%) [14], but was lower than the measure reported by Akay et al. (16.5%) [23]. The difference in the prevalence of SSCD could be due to such reasons as a difference in the studied populations and the slice thickness used for evaluation. The lower the slice thickness, the greater the SSCD prevalence would be. The slice thickness was 0.4 mm in Akay's study, but it was 1 mm in the current study and the one performed by Kurt et al. [14]. The impact of slice thickness on the prevalence of SSCD is recommended to be assessed in future investigations.

A literature review has supported the relationship between the presence of SSCD and anatomical irregularity of the middle cranial fossa. This concomitancy is caused by either a systemic condition or a particular local bone loss [18]. Former studies revealed an association between SSCD and the dehiscence of the tegmen tympani (TT) [16, 35] Fraile Rodrigo et al. showed that TT and SSC had the same embryological origin, by sharing a common layer of external periosteum [36]. In the study by Crovetto et al., the mean RGF was 0.72 ± 0.57 mm [22]. A very similar average RGF was also obtained in the present study (0.75 ± 0.51 mm). Two studies conducted in 2018 and 2020 confirmed the relationship between the dehiscence of the RGF and SSCD [22, 23], to explain this relationship, Crovetto et al. recommended a connection in the embryological development of these structures [22]. Moreover, Whyte et al. also found a significant interaction outcome of TT and SSC statuses on RGF thickness [37].

Crovetto et al. stated that thinning or thickening of the SSC roof was associated with thinning or thickening of the GF roof. Based on the results, 14.1% of cases with SSCD indicated thinning or dehiscence of the RGF. In addition, there was no case of GF dehiscence without the presence of SSCD [22]. However, the current study results indicated that despite the absence of SSCD, there was dehiscence in the roof of the GF. The difference between the results could be attributed to the low sample size of the study by Crovetto et al. (156 vs. 560). The present study indicated a significant association between the thickness of the bone masking the SSC and RGF. Accordingly, the patients with the dehiscence of the GF roof were 12.93 times more likely to have SSCD compared to those without the GF roof dehiscence. As a result, oral and maxillofacial radiologists and otolaryngologists should be aware that When RGF dehiscence is discovered, SSC statuses should be considered, to rule out associated dehiscence.

## 5. Conclusion

In this study, there was no significant relationship between the thickness of the SSC roof with age and sex. The predominant form of SSC morphology was the normal pattern. Moreover, the prevalence of SSCD was 7.7%, with no significant relationship with sex. Nonetheless, females (aged over 45 years) had a 3.79-fold higher chance for SSCD development compared to younger ones. Dehiscence in the GF was observed in 12.5% of the cases. Interestingly, those with GF dehiscence had a 12.93-fold higher chance for SSCD development. Further studies are suggested to evaluate SSCD among females by taking clinical characteristics such as menopause, osteoporosis, and vitamin D deficiency into account.

## Figures and Tables

**Table 1:** The association between age groups/sex and roof thickness of SSC.

Variable		*N*	Roof thickness of SSC		*p*-value
			Mean (mm)	Std. deviation	
Age (years)	<20	84	0.93	0.41	0.660
	20–29	216	0.94	0.51	
	30–39	136	0.97	0.46	
	40–49	66	0.86	0.45	
	50–59	34	0.93	0.65	
	≥60	24	0.83	0.38	
	Total	560	0.93	0.48	

Sex	Female	316	0.95	0.52	0.419
	Male	244	0.91	0.43	

SSC: superior semicircular canal.

**Table 2:** Distribution of SSC radiological patterns among age groups.

Radiological patterns of SSC		Radiological patterns of SSC				
		Normal	Papyraceous	Thick	Pneumatized	Dehiscence
Age groups	<20	62	6	5	9	2
	20–29	154	10	17	15	20
	30–39	106	8	8	8	6
	40–49	52	3	2	2	7
	50–59	18	4	5	0	7
	≥60	16	3	0	4	1

Total (%)		408 (72.9%)	34 (6.1%)	37 (6.6%)	38 (6.8%)	43 (7.7%)

SSC: superior semicircular canal.

**Table 3:** Comparison of the participants aged below and above 45 years regarding the prevalence of SSCD.

	Age	Count	SSCD		OR(95% CI)	*p*-value
			No	Yes		
Female	≤45	274 (86.7%)	258 (94.2%)	16 (5.8%)	3.79 (1.51–9.53)	0.003
	>45	42 (13.3%)	34 (81.0%)	8 (19.0%)		

Male	≤45	210 (86.1%)	194 (92.4%)	16 (7.6%)	1.17 (0.32–4.26)	0.808
	>45	34 (13.9%)	31 (91.2%)	3 (8.8%)		

SSCD: superior semicircular canal dehiscence; OR: odds ratio; CI: confidence interval.

**Table 4:** The relationship between the presence/absence of SSCD and the dehiscence/thickness of the RGF thickness.

		SSCD		OR (95% CI)	*p*-value
		No	Yes		
RGF	≤1 mm	396 (90.4%)	42 (9.6%)		0.008
	1 < RGF ≤ 2 mm	103 (99%)	1 (1%)	0.09 (0.01–0.67)	
	>2 mm	12 (100%)	0 (0%)	0.90 (0.87–0.93)	

Dehiscence of the GF	No	465 (96.1%)	19 (3.9%)	12.93 (6.59–25.37)	≤0.001
	Yes	46 (65.7%)	24 (34.3%)		

SSCD: superior semicircular canal dehiscence; RGF: roof of the glenoid fossa; GF: glenoid fossa, OR: odds ratio; CI: confidence interval.

## Data Availability

The data used to support the findings of this study are restricted by the Human Ethics Review Committee of Shiraz Dental School (# IR Sums.Dental.REC.1400.034) in order to protect patient privacy. Data are available from archives of the Radiology Department of Shiraz Dental School for researchers who meet the criteria for access to confidential data.

## References

[1] Curtin H., Gupta R., Bergeron T., Som P., Curtin H. (2011). Embryology, anatomy, and imaging of temporal bone. *Medicine (Baltimore)*.

[2] Minor L. B., Solomon D., Zinreich J. S., Zee D. S. (1998). Sound- and/or pressure-induced vertigo due to bone dehiscence of the superior semicircular canal. *Archives of Otolaryngology - Head and Neck Surgery*.

[3] Carey J. P., Minor L. B., Nager G. T. (2000). Dehiscence or thinning of bone overlying the superior semicircular canal in a temporal bone survey. *Archives of Otolaryngology - Head and Neck Surgery*.

[4] Brandolini C., Modugno G. C., Pirodda A. (2014). Dehiscence of the superior semicircular canal: a review of the literature on its possible pathogenic explanations. *European Archives of Oto-Rhino-Laryngology*.

[5] Hirvonen T. P., Weg N., Zinreich S. J., Minor L. B. (2003). High-resolution CT findings suggest a developmental abnormality underlying superior canal dehiscence syndrome. *Acta Oto-Laryngologica*.

[6] Williamson R. (2003). Coronal computed tomography prevalence of superior semicircular canal dehiscence. *Otolaryngology - Head and Neck Surgery*.

[7] Crovetto De La Torre M. A., Whyte Orozco J., Cisneros Gimeno A. I., Basurko Aboitz J. M., Oleaga Zufiria L., Sarrat Torreguitart R. (2005). Superior semicircular canal dehiscence syndrome. Embryological and surgical consideration. *Acta Otorrinolaringológica Española*.

[8] Krombach G. A., Martino E., Martiny S. (2006). Dehiscence of the superior and/or posterior semicircular canal: delineation on T2-weighted axial three-dimensional turbo spin-echo images, maximum intensity projections and volume-rendered images. *European Archives of Oto-Rhino-Laryngology*.

[9] Zhou G., Gopen Q., Poe D. S. (2007). Clinical and diagnostic characterization of canal dehiscence syndrome: a great otologic mimicker. *Otology & Neurotology*.

[10] Cloutier J. F., Bélair M., Saliba I. (2008). Superior semicircular canal dehiscence: positive predictive value of high-resolution CT scanning. *European Archives of Oto-Rhino-Laryngology*.

[11] Sequeira S. M., Whiting B. R., Shimony J. S., Vo K. D., Hullar T. E. (2011). Accuracy of computed tomography detection of superior canal dehiscence. *Otology & Neurotology*.

[12] Tavassolie T. S., Penninger R. T., Zuñiga M. G., Minor L. B., Carey J. P. (2012). Multislice computed tomography in the diagnosis of superior canal dehiscence: how much error, and how to minimize it?. *Otology & Neurotology*.

[13] Eibenberger K., Carey J., Ehtiati T., Trevino C., Dolberg J., Haslwanter T. (2014). A novel method of 3D image analysis of high-resolution cone beam CT and multi slice CT for the detection of semicircular canal dehiscence. *Otology & Neurotology*.

[14] Kurt H., Orhan K., Aksoy S., Kursun S., Akbulut N., Bilecenoglu B. (2014 Mar). Evaluation of the superior semicircular canal morphology using cone beam computed tomography: a possible correlation for temporomandibular joint symptoms. *Oral Surgery, Oral Medicine, Oral Pathology and Oral Radiology*.

[15] Brantberg K., Bergenius J., Mendel L., Witt H., Tribukait A., Ygge J. (2001). Symptoms, findings and treatment in patients with dehiscence of the superior semicircular canal. *Acta Oto-Laryngologica*.

[16] Crovetto M., Whyte J., Rodriguez O. M., Lecumberri I., Martinez C., Elexpuru J. (2010 Nov). Anatomo-radiological study of the superior semicircular canal dehiscence. *European Journal of Radiology*.

[17] Mondina M., Bonnard D., Barreau X., Darrouzet V., Franco-Vidal V. (2013). Anatomo-radiological study of the superior semicircular canal dehiscence of 37 cadaver temporal bones. *Surgical and Radiologic Anatomy*.

[18] Crovetto M. A., Whyte J., Rodriguez O. M. (2012). Influence of aging and menopause in the origin of the superior semicircular canal dehiscence. *Otology & Neurotology*.

[19] Davey S., Kelly-Morland C., Phillips J. S., Nunney I., Pawaroo D. (2015). Assessment of superior semicircular canal thickness with advancing age. *The Laryngoscope*.

[20] Mahulu E. N., Fan X., Ding S. (2019). The variation of superior semicircular canal bone thickness in relation to age and gender. *Acta Oto-Laryngologica*.

[21] Yu A., Teich D. L., Moonis G., Wong E. T. (2012). Superior semicircular canal dehiscence in East Asian women with osteoporosis. *BMC Ear Nose and Throat Disorders*.

[22] Crovetto-Martínez R., Vargas C., Lecumberri I., Bilbao A., Crovetto-De la Torre M., Whyte-Orozco J. (2018). Radiologic correlation between the thickness of the roof of the glenoid fossa and that of the bony covering of the superior semicircular canal. *Oral Surgery, Oral Medicine, Oral Pathology and Oral Radiology*.

[23] Akay G., Karataş M. S., Karadağ Ö, Üçok C. Ö, Güngör K. (2020). Examination of the possible relation of the superior semicircular canal morphology with the roof thickness of the glenoid fossa and bone changes of the temporomandibular joint. *European Archives of Oto-Rhino-Laryngology*.

[24] Cisneros A. I., Whyte J., Martínez C. (2013). Radiological patterns of the bony roof of the superior semicircular canal. *Surgical and Radiologic Anatomy*.

[25] Dalchow C. V., Knecht R., Grzyska U., Muenscher A. (2013). Radiographic examination of patients with dehiscence of semicircular canals with digital volume tomography. *European Archives of Oto-Rhino-Laryngology*.

[26] Sepulveda I., Schmidt T., Platin E. (2014). Use of cone beam computed tomography in the diagnosis of superior semicircular canal dehiscence. *Journal of Clinical Imaging Science*.

[27] Chilvers G., McKay-Davies I. (2015). Recent advances in superior semicircular canal dehiscence syndrome. *Journal of Laryngology & Otology*.

[28] El Hadi T., Sorrentino T., Calmels M. N., Fraysse B., Deguine O., Marx M. (2012). Spontaneous tegmen defect and semicircular canal dehiscence: same etiopathogenic entity?. *Otology & Neurotology*.

[29] Takahashi N., Tsunoda A., Shirakura S., Kitamura K. (2012). Anatomical feature of the middle cranial fossa in fetal periods: possible etiology of superior canal dehiscence syndrome. *Acta Oto-Laryngologica*.

[30] Stevens S. M., Lambert P. R., Rizk H., McIlwain W. R., Nguyen S. A., Meyer T. A. (2015). Novel radiographic measurement algorithm demonstrating a link between obesity and lateral skull base attenuation. *Otolaryngology - Head and Neck Surgery*.

[31] Nadgir R. N., Ozonoff A., Devaiah A. K., Halderman A. A., Sakai O. (2011). Superior semicircular canal dehiscence: congenital or acquired condition?. *American Journal of Neuroradiology*.

[32] Hagiwara M., Shaikh J. A., Fang Y., Fatterpekar G., Roehm P. C. (2012). Prevalence of radiographic semicircular canal dehiscence in very young children: an evaluation using high-resolution computed tomography of the temporal bones. *Pediatric Radiology*.

[33] Evlice B., Çabuk D. S., Duyan H. (2021 Nov). The evaluation of superior semicircular canal bone thickness and radiological patterns in relation to age and gender. *Surgical and Radiologic Anatomy*.

[34] Karimnejad K., Czerny M. S., Lookabaugh S., Lee D. J., Mikulec A. A. (2016). Gender and laterality in semicircular canal dehiscence syndrome. *Journal of Laryngology & Otology*.

[35] Nadaraja G. S., Gurgel R. K., Fischbein N. J. (2012). Radiographic evaluation of the tegmen in patients with superior semicircular canal dehiscence. *Otology & Neurotology*.

[36] Fraile Rodrigo J. J., Cisneros A. I., Obón J. (2016). Ontogenetic explanation for tegmen tympani dehiscence and superior semicircular canal dehiscence association. *Acta Otorrinolaringologica (English Edition)*.

[37] Whyte J., Cisneros A. I., Fraile J. J. (2020). Interaction effect of tegmen tympani and superior semicircular canal statuses on the thickness of the roof of the glenoid fossa: a cross-sectional descriptive study. *Surgical and Radiologic Anatomy*.

